# Synteny-Based Development of CAPS Markers Linked to the *Sweet kernel* LOCUS, Controlling Amygdalin Accumulation in Almond (*Prunus dulcis* (Mill.) D.A.Webb)

**DOI:** 10.3390/genes9080385

**Published:** 2018-07-31

**Authors:** Francesca Ricciardi, Jorge Del Cueto, Nicoletta Bardaro, Rosa Mazzeo, Luigi Ricciardi, Federico Dicenta, Raquel Sánchez-Pérez, Stefano Pavan, Concetta Lotti

**Affiliations:** 1 Department of the Sciences of Agriculture, Food and Environment, University of Foggia, via Napoli 25, I-71100 Foggia, Italy; francesca.ricciardi@unifg.it (F.R.); concetta.lotti@unifg.it (C.L.); 2 Agroscope Forschungszentrum Conthey, Route des Eterpys 18, 1964 Conthey, Switzerland; jorge-luis.del-cueto-chocano@agroscope.admin.ch; 3 Department of Soil, Plant and Food Science, University of Bari “Aldo Moro”, Via Amendola 165/A, I-70126 Bari, Italy; niclab86@hotmail.com (N.B.); rosa.mazzeo@uniba.it (R.M.); luigi.ricciardi@uniba.it (L.R.); 4 Department of Plant Breeding, CEBAS-CSIC, 30100 Espinardo, Murcia, Spain; fdicenta@cebas.csic.es; 5 Plant Biochemistry Laboratory, Faculty of Science, University of Copenhagen, DK-1871 Copenhagen C, Denmark; 6 VILLUM Research Center for Plant Plasticity, DK-1871 Frederiksberg C, Denmark; 7 Institute of Biomedical Technologies, National Research Council (CNR), Via Amendola 122/D, I-70126 Bari, Italy

**Keywords:** almond, plant breeding, mapping, molecular markers, kernel taste, *Sk* locus

## Abstract

The bitterness and toxicity of wild-type seeds of *Prunoideae* is due to the cyanogenic glucoside amygdalin. In cultivated almond (*Prunus dulcis* (Mill.) D.A. Webb), a dominant mutation at the *Sk* locus prevents amygdalin accumulation and thus results in edible sweet kernels. Here, we exploited sequence similarity and synteny between the genomes of almond and peach (*Prunus persica* (L.) Batsch) to identify cleaved amplified polymorphic sequence (CAPS) molecular markers linked to the *Sk* locus. A segregant F_1_ population was used to map these markers on the *Sk* genomic region, together with *Sk*-linked simple sequence repeat (SSR) markers previously described. Molecular fingerprinting of a cultivar collection indicated the possibility to use CAPS polymorphisms identified in this study in breeding programs arising from different parental combinations. Overall, we highlight a set of codominant markers useful for early selection of sweet kernel genotypes, an aspect of primary importance in almond breeding. In addition, by showing collinearity between the physical map of peach and the genetic map of almond with respect to the *Sk* genomic region, we provide valuable information for further marker development and *Sk* positional cloning.

## 1. Introduction

Wild-type seeds of *Prunoidaeae* are bitter and toxic, as they accumulate amygdalin, a cyanogenic phytoanticipin, thought to play an important role in defense against herbivores and pathogens [1]. The sweet taste of kernels of cultivated almond (*Prunus dulcis* (Mill.) D.A. Webb syn. *Prunus amygdalus* Batsch) likely originates from a mutation selected during domestication and maintained in modern cultivars [2]. Previous genetic analyses have shown that kernel taste in almond depends on the genotype of the mother plant, regardless of the pollinizer [3]. Moreover, this trait is under monogenic control, with the allele conferring sweetness (*Sk*) being dominant over the one conferring bitterness (*sk*) [4,5]. The molecular nature of the *Sk* locus is still unknown, although it was proposed that the *Sk* gene might encode a prunasin hydrolase (PH), converting prunasin, the amygdalin precursor, to glucose and mandelonitrile [6]. Consistently with this hypothesis, the activity and localization of two almond PH proteins, PH691 and PH692, are different in sweet and bitter almond genotypes [7].

DNA markers linked to economically important traits are routinely used to replace or complement traditional phenotypic selection in plant breeding programs. Simple sequence repeat (SSR) markers, revealing DNA length polymorphism at microsatellite loci, are widely used to investigate genetic diversity in plants [8]. Cleaved amplified polymorphic sequence (CAPS) markers, based on the digestion of PCR-amplified fragments, enable the capture of the most common source of DNA variation, i.e., single nucleotide polymorphisms (SNPs), with common lab equipment and without the need of sequencing technologies [9,10,11].

Phenotypic selection of sweet kernel individuals can only be performed after two-three years, due to the vegetative juvenile period of almond. Therefore, in order to aid breeding activities, a number of studies addressed the identification of molecular markers linked to the *Sk* gene (e.g., [4,12]). In one of them, a number of SSR markers on the almond linkage group (LG) 5 was shown to be linked to the *Sk* locus, and two maps for the *Sk* region were reported, one for each of the two parents of a F_1_ mapping population [12].

The genome sequence of peach (*Prunus persica* L.), a species evolutionarily close to almond, has been released (the International Peach Genome Initiative 2013) and is publicly available. In addition, comparative mapping of *Prunus* diploid species indicated high levels of synteny among their genomes [13]. In this study, we exploited sequence similarity and synteny between the peach and almond genomes to identify CAPS molecular markers linked to the *Sk* locus and suitable for marker-assisted breeding programs. Genotyping of a segregating F_1_ population highlighted full collinearity between peach and almond with respect to the *Sk* region, thus providing valuable information for the development of further *Sk*-linked molecular markers and *Sk* positional cloning. 

## 2. Materials and Methods

### 2.1. Plant Material and DNA Isolation

The F_1_ segregating population used in this study originates from the cross between the parental genotypes R1000 (R) and Desmayo Largueta (D) (R x D), both heterozygous at the *Sk* locus. Besides the 167 individuals genotyped by Sánchez-Pérez et al. [12], 134 individuals obtained from crosses carried out in 2009 and 2010 were used in the present study. All the plants are maintained at the experimental orchard of the CEBAS-CSIC (Centro de Edafología y Biología Aplicada del Segura, Consejo Superior de Investigaciones Científicas) in Murcia, Spain.

A collection of 25 almond cultivars from different geographical origin (Table 1) was also genotyped in this study. All the entries are maintained at CEBAS-CSIC.

For each genotype, DNA was extracted from about three young leaves of an individual plant, according to a cetyltrimethylammonium bromide (CTAB) method [14]. DNA quality and concentration were inferred by gel electrophoresis.

### 2.2. Simple Sequence Repeat Marker Analysis

The SSR markers UDA045 and CPDCT028 were obtained using the primer pairs reported by Dirlewanger et al. [15] and Mnejja et al. [16] and an annealing temperature (T_a_) of 56 °C. Fragment length polymorphisms were visualized on 2% agarose gel. The SSR markers EPDCU2584 and BPPCT037 were obtained according to the protocol described by Schuelke [17]. In detail, forward primers reported by Dirlewanger et al. [15] were tagged by adding a 5′ tail with the universal primer M13. PCR reactions were carried out with a T_a_ of 56 °C, using 0.032 µM forward primer, 0.16 µM reverse primer, and 0.08 µM of a primer complementary to the M13 tail and labeled with FAM or HEX fluorescent dyes (Applied Biosystems, Foster City, CA, USA). Amplicons were separated by capillary electrophoresis via automated sequencer (ABI-3500 Genetic Analyzer, Applied Biosystem, Foster City, CA, USA).

### 2.3. Development of Sk-Linked CAPS Markers

Primer sequences of the markers BPPCT037, CPDCT028, EPDCU2584, and UDA045, previously reported to be in linkage with the *Sk* locus [12], were used for a BLAST search against the *Prunus persica* v1.0 genome assembly available at the Genomic Database of Rosaceae (GDR, www.rosaceae.org). Best matching hits delimited a peach genomic region containing several predicted gene sequences, which were used to design several primer pairs with the Primer3 software [18]. PCR were carried out on the two parental genotypes R and D (T_a_ = 58 °C), and amplicons were next purified and sequenced (Macrogen Europe, The Netherlands). Visual analysis of Sanger electropherograms allowed for the distinguishing of heterozygous nucleotide sites segregating in the F_1_ mapping population. Corresponding polymorphisms were used to choose restriction enzymes suitable for the obtainment of CAPS markers, using the CAPS designer tool available at the Sol Genomics Network database [19]. Markers were scored by digesting 5 µL of amplification products with 5 U of the appropriate restriction enzyme and the recovery of cleaved polymorphisms on 2.5% agarose gel.

### 2.4. Linkage Analysis

Simple Sequence Repeat and CAPS markers were tested on the R x D F_1_ population. Phenotyping was carried out by tasting three kernels from each F_1_ individual. The Monte Carlo Maximum Likelihood algorithm implemented by the software JoinMap 4.0 [20] was used to analyze data and develop a genetic map of the *Sk* region.

### 2.5. CAPS Assay on a Cultivar Collection

DNA of the cultivar collection reported in Table 1 was genotyped with the polymorphic CAPS markers used in this study, as described above. For each marker, the polymorphic information content (PIC) was calculated with PICcalc [21].

### 2.6. Bioinformatic Characterization of the Peach Sk Synthenic Region

Information on peach genes localized in a region synthenic to the one containing the *Sk* locus was retrieved by using the GBrowse option available at GDR for the *Prunus persica* v1.0 genome assembly. Putative functions of corresponding proteins were inferred by information available at the InterPro database [22].

## 3. Results

### 3.1. Development of Sk-Linked CAPS Markers

**Figure 1:**
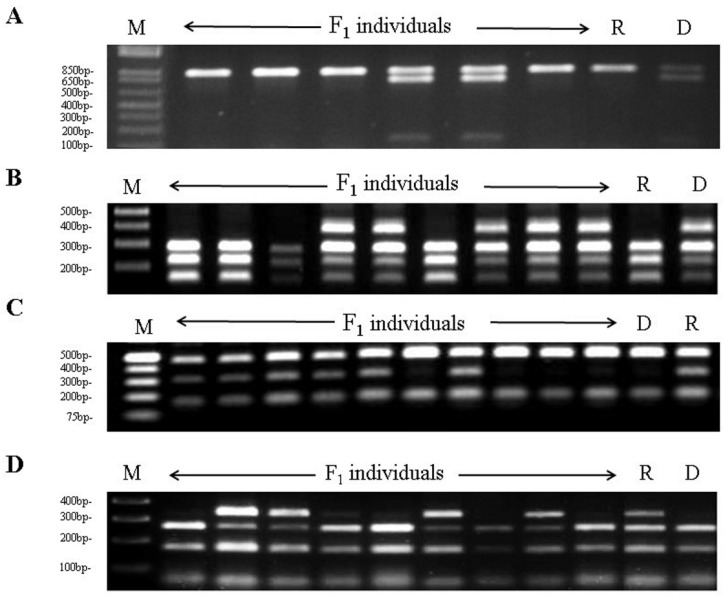
Agarose gel electrophoresis of cleaved amplified polymorphic sequence (CAPS) markers on the two parental genotypes R1000 (R) and Desmayo Largueta (D) and segregating F_1_ individuals. The panels (**A**–**D**) refer to the markers ppa001838m/*Msp*I, ppa006282m/*HpyCH4*V, ppa027182m/*Hpy188*I, and ppa018792m/*Alu*I, respectively.

High level of sequence similarity was reported to occur among *Prunus* genomes [13]. Therefore, a BLAST search was performed to retrieve peach sequences orthologous to the SSR markers BPPCT037, CPDCT028, EPDCU2584, and UDA045, previously shown to be linked to the *Sk* locus on the almond LG5 [12]. Notably, best BLAST hits of these markers all localized on peach scaffold 5. Next, high level of synteny between almond and peach in the *Sk* genomic region was assumed to develop new markers linked to the *Sk* region. In more detail, primer pairs were designed on several peach genes included in the 810 Kb interval flanked by the orthologous sequences of UDA045 and CPDCT028, previously reported to delimit the *Sk* locus [12]. In addition, a primer pair was designed on a gene mapping just outside the same interval and encoding a sugar transporter, named ppa018792m at the GDR database. This transporter, if involved in prunasin translocation, could have explained specific accumulation of prunasin only in the tegument of bitter almonds [7]. Sequence analysis of PCR products revealed the presence of four SNPs segregating in the R x D mapping population, and this information was used to develop CAPS markers named on basis of the peach genes they refer to (ppa001838m, ppa006282m, ppa027182m, and ppa018792m) and the restriction enzyme used to reveal polymorphism (Table 2 and Figure 1).

### 3.2. *Sk* Mapping and Syntenic Relationships with the Peach Genome

**Figure 2:**
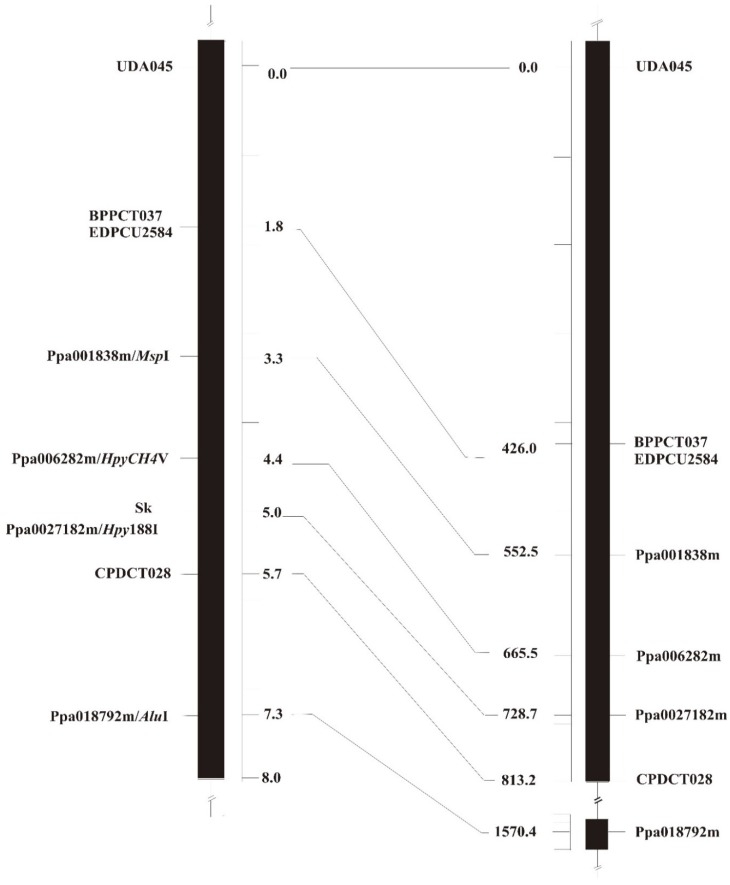
Syntenic relationship between the almond *Sk* linkage map (**left**) and the peach physical map (**right**) in the *Sk* genomic region.

Newly identified CAPS markers and previously reported SSR markers were used to fingerprint a mapping population of 301 individuals, originating from the parental lines R and D. The population included the 167 mapping individuals genotyped in the previous work of Sánchez-Pérez et al. [12] and additional 134 individuals. Marker data were merged with phenotypic scores and used to produce a map of the *Sk* region. Notably, the order of markers on almond LG5 is fully in accordance with the order of marker ortholog sequences on peach scaffold 5 (Figure 2), indicating collinearity between the two genomes. Co-segregation was found for the marker pair BBCT037/EPDCU2584. In addition, the marker ppa027182m/*Hpy188I* co-segregated with the *Sk* locus. The markers ppa006282m/*HpyCH4*V and CPDCT028 were mapped on the two sides of the *Sk* locus, at distances of 0.6 and 0.7 cM, respectively (Figure 2). This corresponds, in peach, to a physical genomic interval of about 151 Kb, spanning twenty predicted coding sequences (Table 3).

### 3.3. Marker Validation in An Almond Germplasm Collection

In order to be useful in breeding programs, markers should highlight polymorphism in different combinations of parental genotypes. Therefore, we tested the CAPS markers identified in this study on an almond germplasm collection of 25 cultivars of different geographical origin (Table 1). All the markers highlighted polymorphism. The polymorphic information content (PIC) associated with each marker were 0.04 for ppa001838m/*Msp*I, 0.37 for ppa006282m/*HpyCH4*V, 0.26 for ppa027182m/*Hpy188*I and 0.18 for ppa018792m/*Alu*I.

## 4. Discussion

The selection of sweet kernel genotypes is a major task in almond breeding programs, which often involve populations segregating at the *Sk* locus. Phenotypic selection for kernel taste is costly and time-consuming, as this trait is manifested only after a long juvenile phase, thus marker-assisted selection is highly desirable. In this study, we exploited sequence similarity and synteny between the peach and almond genomes to identify four co-dominant CAPS markers highly predictive of kernel taste. Indeed, by the analysis of a large F_1_ population, these markers displayed either co-segregation or tight linkage (0.6, 1.7 and 2.3 cM) with the *Sk* locus. Except for ppa001838m/*Msp*I, the other three CAPS markers reported here were associated with good PIC estimates (0.18, 0.26 to 0.37) when tested on a germplasm collection of 25 individuals, thus indicating that they are suitable to reveal polymorphism in different breeding populations. Concerning technical aspects, all the CAPS markers identified in this study are based on agarose gel electrophoresis, so they can be easily obtained with relatively inexpensive laboratory equipment. However, the same SNP polymorphisms used here for CAPS marker development might be conveniently used to set up marker assays which are more suitable to automation, such as high resolution melting (HRM) and competitive allele-specific polymerase chain reaction [10,23].

Prior to this study, Sánchez-Pérez et al. [12] also performed a linkage analysis of the *Sk* genomic region, using a panel of SSR molecular markers and a F_1_ population of 167 individuals originating from the two parental genotypes R1000 and Desmayo Largueta. However, the two maps obtained in that work, one for each parent, displayed some inconsistencies regarding the position of common markers, and their distance from the *Sk* locus. Here, *de novo* genotyping of four of these SSR markers (BPPCT037, CPDCT028, EPDCU2584, and UDA045) on the same F_1_ individuals resulted in the detection of a few scoring errors. Most of them refer to the marker BPPCT037, harboring a length polymorphism of a single base pair (131/132) in the parental genotype R1000. Calling these two alleles was straightforward in our hands, as we used capillary electrophoresis on automated sequencer, whereas it was troublesome in the work of Sánchez-Pérez et al. [12], which made use of polyacrylamide gel electrophoresis. The accuracy of our map was further improved by increasing the size of the mapping population with additional 134 individuals. 

Importantly, the order of markers in the genetic map provided in this study is fully consistent with the one of ortholog sequences in the peach physical map (Figure 2), thus corroborating the notion that genomes across the *Prunus* genus are collinear [13]. Based on our data, the *Sk* gene is likely located in a genomic interval flanked by two markers ppa006282m/*HpyCH4*V and CPDCT028, corresponding in peach to a physical region of about 151 Kb. Notably, none of the twenty genes included in this interval encodes proteins known to play an obvious role in the biosynthesis or degradation of cyanogenic glucosides, including prunasin hydrolases, which were previously hypothesized to be the product of the *Sk* gene [6]. The presence, in the same region, of several putative helix-loop-helix proteins might suggest that amygdalin accumulation is controlled at the transcriptional level.

Overall, this study provides valuable tools to aid the selection of sweet kernel genotypes in almond breeding programs and lays a foundation for the isolation of the *Sk* gene via positional cloning. Aiming to further delimit the *Sk* genomic region, we are currently working at the development of new F_1_ individuals and *Sk*-linked molecular markers.

## Figures and Tables

**Table 1:** Panel of cultivars genotyped in this study.

Cultivar	Origin
Del Cid	Spain
Ramillete	Spain
Atocha	Spain
Desmayo Largueta	Spain
Marcona	Spain
Vivot	Spain
Peraleja	Spain
Antoñeta	Spain
Ferragnès	France
Lauranne	France
Marta	Spain
R-1000	France
Mono	USA
Tioga	USA
Titan	USA
Wawona	USA
Nonpareil	USA
Tardy-Nonpareil	USA
Achaak	Tunisia
Ardechoise	France
Chellaston	Australia
Primorskii	Russia
Garrigues	Spain
Genco	Italy
Tuono	Italy

**Table 2:** Features of the cleaved amplified polymorphic sequence (CAPS) markers developed in this study include the following: primer sequences, estimated length of PCR product, single nucleotide polymorphisms (SNP) generating differential cleavage, and estimated length of digestion products in the parental genotypes Desmayo Largueta (D) and R1000 (R).

Marker	Primer Sequences (5′-3′)	PCR Product (bp)	SNP	Digestion Products (bp)
ppa001838m/*Msp*I	F: GGTTGTTCTGGGAGATGGAA R: ACTTGACCGCAACCAAAATC	800	T→G	D: 800, 650, 150
				R: 800
ppa006282m/*HpyCH*4V	F: GTTTCGCTCGATTGGGTCTC R: ATCATTTCCCGCCTGAATGC	700	G→A	D: 400, 300, 250, 150
				R:300, 250, 150
ppa027182m/*Hpy188*I	F: AAAGAAGATTGGGGCCTTGT R: TGGTTAAGCTTCTCGCGTCT	600	C→T	D: 450, 150
				R: 450, 300, 150,
ppa018792m/*Alu*I	F: ACGTTGTCTCGTTCGTGGTT R: AGGTGCTGCAAAGACACTGA	540	T→C	D:280, 180, 80
				R:340, 280, 180, 80

**Table 3:** Features of peach genes included in the physical interval delimited by ppa006282m and CPDCT028.

GDR ID	Interval on Scaffold 5	InterPro Putative Function
ppa006282m	12.547.702-12.551.295	Uncharacterised protein family UPF0017, hydrolase-like, conserved site
ppa005470m	12.555.940-12.558.349	Cys/Met metabolism, pyridoxal phosphate-dependent enzyme
ppa003882m	12.562.194-12.564.053	Cytochrome P450
ppa011942m	12.576.856-12.578.103	Mediator complex, subunit Med10
ppa023406m	12.587.426-12.589.276	Glyoxal oxidase
ppa022201m	12.597.330-12.598.918	Helix-loop-helix DNA-binding-Transcription factor MYC/MYB
ppa025417m	12.603.688-12.605.325	Helix-loop-helix DNA-binding-Transcription factor MYC/MYB
ppa027182m	12.612.821-12.614.522	Helix-loop-helix DNA-binding-Transcription factor MYC/MYB
ppa015634m	12.625.785-12.627.695	Helix-loop-helix DNA-binding-Transcription factor MYC/MYB
ppa005343m	12.636.946-12.638.591	Helix-loop-helix DNA-binding-Transcription factor MYC/MYB
ppa005388m	12.644.406-12.646.982	Alpha/beta hydrolase fold-1
ppa021506m	12.649.801-12.651.659	GDSL lipase
ppa010428m	12.662.679-12.664.291	Domain of unknown function DUF4033
ppa004653m	12.666.017-12.668.186	Glycoside hydrolase, family 9
ppa005847m	12.669.378-12.670.953	Transmembrane receptor, eukaryota
ppa022759m	12.673.586-12.675.114	Unknown
ppa019752m	12.677.592-12.679.579	WRC domain protein
ppa021141m	12.680.973-12.682.964	IQ motif, EF-hand binding site
ppa019815m	12.687.460-12.688.443	Glutaredoxin
ppa006801m	12.695.757-12.697.169	No apical meristem (NAM) protein
CPDCT028	12.699.037-12.699.598	
